# Neo Left Main Channel Creation Using Double Stenting Alongside a Sapien 3 Aortic Valve Bioprosthesis for Left Main Coronary Obstruction Following Valve-in-Valve Transcatheter Aortic Valve Replacement: A Case Report With Review of Literature

**DOI:** 10.1177/2324709618767696

**Published:** 2018-03-30

**Authors:** Apurva D. Patel, Thomas Haldis, Kais Al Balbissi, Timir Paul

**Affiliations:** 1 University of North Dakota, Fargo, ND, USA; 2 East Tennessee State University, Johnson City, TN, USA; 3 University of Nebraska Medical Center, Omaha, NE, USA

**Keywords:** transcatheter aortic valve replacement, valve-in-valve, left main coronary obstruction, aortic stenosis

## Abstract

Transcatheter aortic valve replacement in the setting of failed surgical bioprosthesis (valve-in-valve) is a valuable option for patients with bioprosthetic aortic stenosis or regurgitation who are deemed high risk for repeat open heart surgery. Although the procedure is successful with proper preprocedural assessment, instances of left main (LM) coronary artery ostium obstruction have been documented. We present a case of LM coronary obstruction in the immediate postoperative period following implantation of a 20-mm Edwards Sapien 3 valve inside the degenerated 21-mm Mitroflow bioprosthesis stenosis, which was treated with double stenting alongside the Edwards Sapien 3 valve creating a channel (“neo left main”) that extended from mid-LM to the upper margin of the Edwards Sapien 3 valve. Although valve-in-valve in a Mitroflow degenerated bioprosthesis is a relatively safe procedure, 2 or more stents may be necessary to scaffold a channel to the coronary arteries between Edwards Sapien 3 prosthesis and aorta in the event of a coronary obstruction.

## Introduction

With the advancement in technology, transcatheter aortic valve replacement (TAVR) for failed surgical aortic bioprosthesis (valve-in-valve [VIV]) has become a widely accepted option for patients at high risk for redo open heart surgery.^1^ The TAVR procedure is successful in the majority of patients, though coronary ostial obstruction has been reported as one of the most serious complications associated with high mortality.^1-5^ Data suggest that insertion of Edwards Sapien 3 valve in VIV procedure is not associated with coronary obstruction.^6-10^ We reported the first case of left main (LM) coronary ostium obstruction following insertion of Edwards Sapien 3 valve on degenerated Mitroflow aortic bioprosthesis stenosis, successfully treated with the insertion of 2 bare metal stents creating a channel between Edwards Sapien 3 valve and aorta toward LM coronary artery ostium.

## Case Description

**Figure 1.**
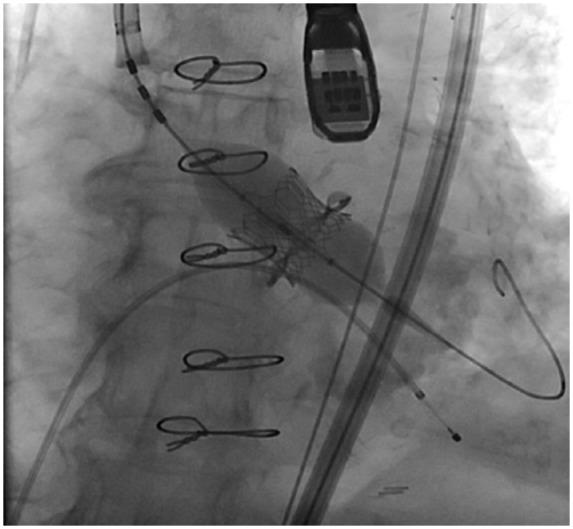
Transesophageal echocardiography guided implantation of a 20-mm Edwards Sapien 3 valve inside a 21-mm Mitroflow bioprosthesis.

**Figure 2.**
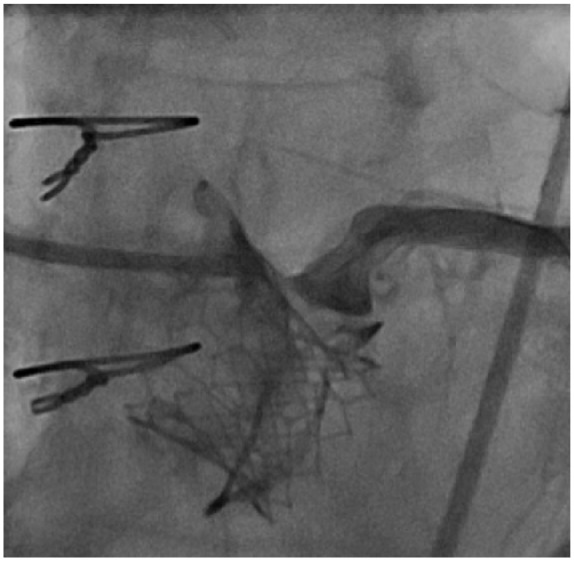
Coronary angiography showing Mitroflow leaflet overriding the left main coronary ostium with poor flow in left coronary artery.

**Figure 3.**
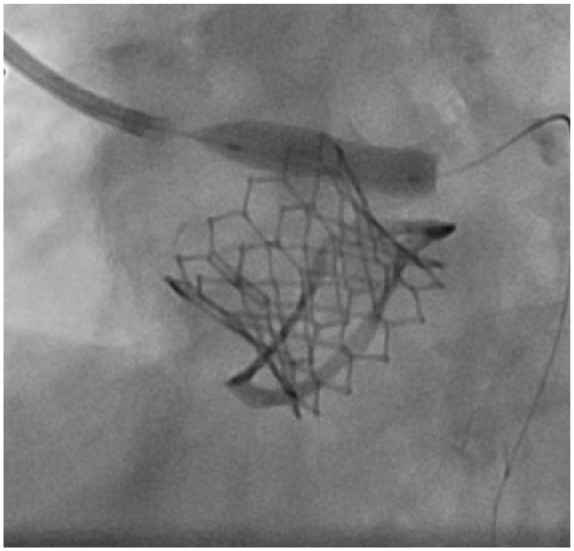
Insertion of left main coronary wire and balloon behind the Sapien 3 valve following inability to cross the upper cells of Edwards Sapien 3 valve due to obstruction by the Mitroflow bioprosthetic valve leaflet.

**Figure 4.**
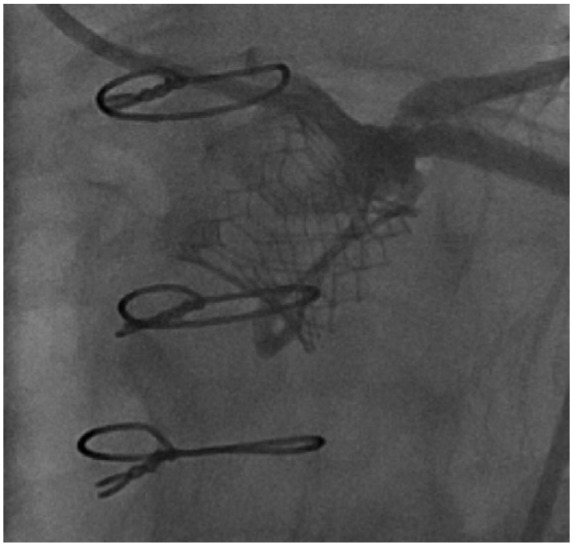
Two bare metal stents effectively scaffolding the Edwards Sapien 3 valve and providing a new channel towards the left main coronary artery.

An 81-year-old female presented with increasing shortness of breath with exertion for about 1 year. Past surgical history was significant for aortic valve replacement using a 21-mm Mitroflow bioprosthesis for aortic stenosis and coronary artery bypass surgery with a left internal mammary artery graft to the left anterior descending artery 3 years before the current presentation. Comorbidities included atrial fibrillation, obesity, hypertension, and hyperlipidemia. Physical examination showed grade 3/6 systolic murmur in the right second intercostal space. Echocardiography revealed severe bioprosthetic stenosis with a mean transaortic gradient of 41 mm Hg and aortic valve orifice of 0.9 cm^2^. The left ventricular systolic function was preserved and there was mild mitral stenosis. Coronary angiography showed a left dominant circumflex and atretic left internal mammary artery graft to the distal left anterior descending artery. LM coronary artery was widely patent. Transfemoral implantation of a 20-mm Edwards Sapien 3 valve inside 21-mm Mitroflow bioprosthesis under the guidance of transesophageal echocardiography was initially uneventful (Figure 1). Postprocedure, the patient was extubated in the catheterization suite, during which she complained of chest pain. Deep ST-segment depression was noted on a cardiac monitor and an electrocardiogram (ECG) suggesting possible anterolateral and inferior subendocardial ischemia. Stat echocardiography showed a mild reduction in left ventricular systolic function, but no evidence of pericardial effusion, aortic root dissection, or aortic hematoma. Urgent coronary angiography revealed Mitroflow leaflet overriding the LM ostium with poor flow in the left coronary artery (Figure 2). Upper cells of Edwards Sapien 3 valve could not be crossed because of obstruction by the prosthetic valve leaflet, so the LM artery was wired and ballooned behind the Sapien 3 valve (Figure 3). A channel (“neo left main”) was created alongside the Edwards Sapien 3 valve extending from mid-LM artery to the upper margin of the Edwards Sapien 3 valve. The first stent was compressed by the Edwards Sapien 3 valve and aorta. Two bare metal stents were needed to scaffold the Edwards Sapien 3 valve efficiently and to provide a new LM coronary artery channel (Figure 4). Following the intervention, the patient’s hemodynamics and ECG changes improved. Repeat echocardiography demonstrated normal ejection fraction and normal valve function. The patient was discharged on postoperative day 4.

## Discussion

Coronary obstruction following VIV TAVR procedure is uncommon with an overall reported incidence of ≤3.5% across different VIV registries.^3,11,12^ This in addition to the data from case reports and case series have documented instances of coronary obstruction with the use of various transcatheter heart valves (THVs) on stented or stentless surgical bioprosthesis valves^3,11-17^ (Table 1). However, no data exist on incidence or management of coronary obstruction in patients undergoing Edwards Sapien 3 THV for the failed Mitroflow surgical bioprosthesis. Mechanical obstruction of LM coronary ostium is more common than right ostium, and acute hemodynamic compromise is reported more often than delayed presentation following TAVR.^3,13-19^ This case highlights the importance of management of coronary obstruction in the setting of VIV utilizing Edwards Sapien 3 THV for the failed Mitroflow bioprosthesis with the insertion of stents between the surgical valve leaflets and aorta creating a channel (“neo left main”) such that the flow through coronary ostium into the coronary system can be regained.

**Table 1.** Coronary Obstruction Following Valve-in-Valve Transcatheter Aortic Valve Replacement.

Author, Year	Type of Study	Number of Subjects	Number of Subjects With Coronary Obstruction, n (%)	Branch of Coronary Ostial Obstruction	Treatment of Coronary Obstruction	Degeneration Mode (Stenosis, Regurgitation, Mixed), n (%)	Type of Surgical Bioprosthesis, n (%)	Type of VIV THV
Webb et al,^11^ 2017	PARTNER 2 VIV registry	365	3 (0.8)	Not available	Not available	Stenosis: 197/357 (55)	Stented: 337/365 (92)	23- or 26- mm Sapien XT
						Regurgitation: 84/357 (24)	Stentless or homograft:22/365 (6)	
						Mixed: 76/357 (21)	Unknown: 6/365 (2)	
Alnasser et al,^12^ 2017	VIV international data registry	162; Portico (n = 54) and CoreValve (n = 108)	2 (1.9); CoreValve	Not available	Not available	Stenosis: 56/162 (35)	Stented: 142/162 (88)	23-, 25-, or 26- mm Portico, or CoreValve
						Regurgitation: 52/162 (32)	Stentless: 20/162 (12)	
						Mixed: 54/162 (33)		
Cokburn et al,^13^ 2017	Case series	6	2	Left main	Subject 1: balloon aortography and repeat surgery	Stenosis: 5 (83)	Sorin Freedom Solo (stentless)	26-mm CoreValve (n = 1); 23-mm Evolut-R (n = 3); 23-mm Lotus (n = 1)
					Subject 2: CoreValve snaring into aorta	Regurgitation: 1 (17)		
Praz et al,^14^ 2016	Case report	1	1	Left and right main	Removal of Lotus valve	Regurgitation	Sorin Freedom Solo (stentless)	29-mm CoreValve Evolut-R
Fabris et al,^15^ 2016	Case report	1	1	Left and right main	Resolute stent 4.0 × 15 mm in left main, and 3.5 × 18 mm in right main artery	Regurgitation	St. Jude Toronto (stentless)	29-mm CoreValve
Allende et al,^16^ 2014	Case report	1	1	Left main	Promus element 4.0 × 12 mm stent	Mixed	Freestyle (stentless)	23-mm Sapien XT
Dvir et al,^3^ 2012	Global VIV registry	202; CoreValve (n = 124) and Sapien XT (n = 78)	7 (3.5); 4 CoreValve and 3 Sapien XT	Left main	Not available	Stenosis: 85/202 (42)	Stented: 155 (77)	26-mm CoreValve and 23-mm Sapien XT
						Regurgitation: 68/202 (34)	Stentless: 47 (23)	
						Mixed: 49/202 (24)		
Gurvich et al,^17^ 2011	Case series	2	2	Left and right main (subject 1); left main (subject 2)	CABG and sternotomy (subject 1); inoperable (subject 2)	Mixed	Mitroflow (stented)	23-mm Edwards Sapien (subject 1) and 26-mm CoreValve (subject 2)

Abbreviations: VIV, valve-in-valve; THV, transcatheter heart valve; PARTNER 2, placement of aortic transcatheter valves 2; CABG, coronary artery bypass grafting.

Risk of coronary obstruction following VIV procedure depends on various factors such as the type of bioprosthetic valve used during the initial valve surgery (ie, stentless vs internally stented surgical valves, supra-annular position, high-leaflet profile, and bulky leaflets), anatomical factors (ie, low-lying coronary ostia, narrow sinotubular junction, narrow sinuses of Valsalva, and previous root repair) as well as THV factors (ie, extended sealing cuff and high implantation).^5^ According to the literature, it has been postulated that the stentless or internally stented valves such as Mitroflow may pose a higher risk of coronary obstruction following VIV procedure. This is likely due to the extension of the leaflets of the Mitroflow valve in an outward direction in tubular fashion beyond the surgical device frame such that the leaflets then compress onto the aortic walls and thus obstructing coronary blood flow. Although in registries where Mitroflow was one of the most commonly used valves, in majority of cases the VIV procedures were uneventful.^5^ However, in comparison to the stented valves, Mitroflow was associated with significantly higher proportion of mortality associated with the coronary obstruction (7.7% out of total of 57.1% mortality rate) following VIV procedure in Global VIV Registry (*P* = .049).^6^ Suggested strategies for reduction of coronary obstruction include an initial evaluation with computed tomography, transthoracic and transesophageal echocardiography, and/or fluoroscopy/angiography for precise placement.^5,6^ If VIV is considered an optimal approach in patients with high risk for surgery, then during the procedure it is recommended to perform balloon valvuloplasty initially. If after valvuloplasty a patient remains hemodynamically stable and risk of coronary obstruction seems higher, then it is recommended to preemptively protect the coronary by putting a wire and a stent.^5,20^ Once the valve is deployed, it is recommended to take several angiographic pictures from different directions during withdrawal of guide, such that the assessment of coronary obstruction before removal of wire and stent can be made. Since the late presentation of coronary obstruction is possible, postprocedure ECG, echocardiography, and signs and symptoms of myocardial ischemia evaluation are warranted.^5^ In this report, the patient developed coronary obstruction even after a thorough assessment of the risk of obstruction. However, there are operative factors that could have contributed to the coronary obstruction and with the use of procedural steps as highlighted above could have provided smoother management in this instance. First, the implantation of SAPIEN 3 valve inside a Mitroflow prosthesis was high (Figures 2-4). Second, it would have been the more straightforward management of coronary obstruction if we had wired the coronary ostium before the implantation of the SAPIEN 3 valve. We did not anticipate this problem beforehand and hence did not wire the ostium. In situations such as this, where it is difficult to approach coronary ostium post VIV, creating a channel between obstructing and bulkier Mitroflow leaflets and aorta could be considered such that deployment of stent in coronary ostium becomes easier and such a channel could potentially be kept open from the upper aspect of the Mitroflow leaflets and Edwards Sapien 3 valve to the LM coronary ostium.

## Conclusion

Coronary obstruction is a rare complication of VIV procedure when an Edward Sapien 3 valve is used in the setting of degenerated Mitroflow bioprosthesis. Careful assessment of the anatomical relationship between coronary ostia and Mitroflow bioprosthesis using either computed tomography and/or transesophageal echocardiography is important. Placement of stents between bioprosthetic valve leaflets and aorta may be needed to create a channel between the upper margins of Edward Sapien 3 valve and LM artery in the setting of LM ostium obstruction.
